# Exploring the Needs and Preferences of Users and Parents to Design a Mobile App to Deliver Mental Health Peer Support to Adolescents With Type 1 Diabetes: Qualitative Study

**DOI:** 10.2196/64267

**Published:** 2025-01-20

**Authors:** Titilola I Yakubu, Poonamdeep Jhajj, Samantha Pawer, Nicholas C West, Shazhan Amed, Tricia S Tang, Matthias Görges

**Affiliations:** 1 Experimental Medicine Program Department of Medicine The University of British Columbia Vancouver, BC Canada; 2 Research Institute BC Children’s Hospital Vancouver, BC Canada; 3 MD Undergraduate Program Faculty of Medicine The University of British Columbia Vancouver, BC Canada; 4 Division of Endocrinology & Diabetes Department of Pediatrics The University of British Columbia Vancouver, BC Canada; 5 Division of Endocrinology Department of Medicine The University of British Columbia Vancouver, BC Canada; 6 Department of Anesthesiology, Pharmacology and Therapeutics The University of British Columbia Vancouver, BC Canada

**Keywords:** peer support, type 1 diabetes, digital interventions, diabetes distress, depression, mental health, focus groups, disease management, adolescent, parent, mobile app, mHealth, type 1, diabetes, qualitative study, physical health, psychological, emotional health, mental health support, thematic analysis, data collection, mobile health

## Abstract

**Background:**

Beyond physical health, managing type 1 diabetes (T1D) also encompasses a psychological component, including diabetes distress, that is, the worries, fears, and frustrations associated with meeting self-care demands over the lifetime. While digital health solutions have been increasingly used to address emotional health in diabetes, these technologies may not uniformly meet the unique concerns and technological savvy across all age groups.

**Objective:**

This study aimed to explore the mental health needs of adolescents with T1D, determine their preferred modalities for app-based mental health support, and identify desirable design features for peer-delivered mental health support modeled on an app designed for adults with T1D.

**Methods:**

A semistructured qualitative focus group study was conducted with adolescents with T1D and parents of adolescents with T1D. Data were collected through pre–focus group surveys, including sociodemographic background, diabetes status, health care experiences, and focus group sessions, including their opinions on peer support and technology. A thematic analysis following an inductive and iterative process was performed to develop themes and subthemes from the collected information.

**Results:**

Focus group participants included 10 adolescents (mean 16, SD 1 years; 8/10, 80% female; who had been living with diabetes for an average of 9, SD 5 years) and 10 parents (mean age 51, SD 7 years; 9/10, 90% female). Four core themes emerged: (1) experience: navigating adolescence with T1D, (2) empowerment: support systems that enabled better management of their T1D, (3) obstacles: societal barriers that affect adolescents’ T1D management, and (4) innovation: adolescent-driven preferences for digital peer support platforms.

**Conclusions:**

App-based peer support offers a promising avenue for addressing the mental health needs of adolescents with T1D. Understanding the unique support needs of these adolescents and using this information to suggest design considerations for a mental health peer support app is an important step toward addressing their complex emotional and social challenges.

## Introduction

Managing type 1 diabetes (T1D) extends beyond physical health to include a significant psychological component [1]. This psychological aspect is often due to diabetes distress (DD), a state of emotional burden directly related to the demands of living with diabetes [2]. DD is influenced by various factors, including the complexity of diabetes management, social dynamics, and puberty-related changes (both physical and psychosocial) that occur during adolescence [3]. For example, adolescents may feel shame and stigma from visible self-care tasks, which are sometimes mistaken for illicit drug use, leading to questioning by authority figures and reluctance to engage in public self-care needs [4].

In Canada, accessing psychological support is inconsistent across provinces [5]; therefore, obtaining treatment for DD may pose a significant challenge. Barriers include uncertainties about where to seek help [6], long waiting times [7], a shortage of mental health care professionals [6], concerns about stigma [8], geographic or demographic disparities (impacting youth, rural communities, and Indigenous populations) [6], and the financial burden of services not covered by private insurance plans [5].

Peer support may present a potential solution for adolescents with T1D, especially when facilitated through digital platforms [9]. This age group is highly attuned to technology, often preferring digital interactions over in-person ones [10]. Digital platforms offer the privacy and flexibility that adolescents value, allowing them to seek support without the discomfort or stigma of face-to-face encounters. In addition, these platforms provide the convenience of accessing support at any time and from any location, which is particularly important given the financial and geographic barriers to accessing traditional mental health services [10]. These platforms also allow adolescents with T1D to tailor support to their specific needs and preferences, providing a space to share experiences, express empathy, and exchange bidirectional assistance in managing their condition [9]. However, these digital platforms should be codesigned with the target population to be effective [11].

T1D REACHOUT (The University of British Columbia) is a peer-led mobile app to support mental health, cocreated by researchers and adults with T1D living in British Columbia, Canada [12]. It offers support mechanisms, including (1) one-on-one support through a self-selected peer supporter, (2) group texting support through a 24/7 chat room, and (3) face-to-face group sessions through video huddles. The app is developed using a participatory approach, ensuring that the target population’s preferences and unique challenges were at the forefront of its design. While the direct impact of this participatory approach on the app’s effectiveness requires further empirical validation, the literature on digital health suggests that user engagement in design processes can enhance the relevance and usability of interventions [11,13,14].

Tailoring the T1D REACHOUT app’s functionalities to adolescents with T1D may address some of the dimensions of DD. Focus groups were selected as the primary method for user engagement because they provide a dynamic environment for participants to discuss shared experiences and preferences [15]. The group setting encourages interaction, allowing adolescents to build on each other’s ideas and reveal insights that might not emerge in one-on-one interviews.

Therefore, the aims of this study were three-fold: (1) to explore the mental health needs of adolescents with T1D in British Columbia; (2) to determine their preferred modalities for app-based mental health support delivery; and (3) to identify the desirable design features for a peer-delivered mental health support app for adolescents, using the existing T1D REACHOUT app as a model.

## Methods

### Study Design

We conducted a semistructured qualitative study involving focus groups with a convenience sample comprising either adolescents living with T1D or parents of adolescents with T1D from families receiving care at BC Children’s Hospital (BCCH) or in the Interior Health. Our findings are reported following the COREQ (Consolidated Criteria for Reporting Qualitative Research) checklist [16], given in Multimedia Appendix 1.

### Ethical Considerations

Ethical approval for this study was obtained from the University of British Columbia–Children’s & Women’s Health Centre of British Columbia Research Ethics Board (H21-01806; approval date: January 25, 2022; principal investigator: MG). Additional approvals were secured from the Interior Health Authority (IHA) and Vancouver Island Health Authority (VIHA) research ethics boards.

All participants provided informed consent (some adolescents provided assent with their parent or guardian providing consent, while others consented directly) before participating in the study. Initial consent discussions were conducted through Zoom videoconferencing software (Zoom Video Communications) or telephone to explain the study objectives and address participants’ questions. Informed consent and assent were documented electronically using the REDCap (Research Electronic Data Capture; Vanderbilt University) eConsent [17,18].

All focus group recordings were automatically transcribed by the Zoom videoconferencing software and deidentified before analysis by replacing identifiable information, such as names, with participant IDs. Study data were stored securely, and only study team members could access them.

Participants received a CAD $25 (approximately US $18) e-gift card as a token of appreciation for their time and participation in the focus groups.

### Sampling and Recruitment

Recruitment began in March 2022 and concluded in February 2023. To be eligible, adolescent participants had to meet the following inclusion criteria: (1) being aged 15-18 years; (2) having physician-diagnosed T1D; (3) having access to a smart device or computer; and (4) residing in the IHA region, VIHA region, or receiving care at BCCH. The decision to include diverse locations was made to capture a broader range of perspectives, with the aim of achieving a more comprehensive representation of our participants. For parents or guardians, inclusion criteria were as follows: (1) having a child with T1D aged 15-18 years; (2) having access to a smart device or computer; and (3) residing in British Columbia.

We used diverse recruitment methods: invitation emails were sent from the BCCH Diabetes Clinic to families who had authorized contact for research, and families attending the BCCH Diabetes Clinic were recruited in person; invitation letters were sent from Diabetes Educational Centers in IHA and VIHA to eligible families; and promotional flyers at diabetes clinics, social media posts on T1D-specific Facebook groups, and referrals from pediatric endocrinologists who identified participants likely to benefit from the study were also used.

### Data Collection

#### Prestudy Survey

Participants completed a prestudy survey using the REDCap platform [17,18], administered after the informed consent process. The surveys captured the demographic and care context and the T1D challenges to contextualize the focus group results; the survey data were not analyzed thematically but served as background information only to facilitate focus group stratification and contextualize discussions. These pre–focus group surveys (given in Multimedia Appendices 2 and 3) assessed sociodemographic background, diabetes status, health care experiences, opinions and experiences with peer support, and technological preferences. Parent surveys assessed sociodemographic background, their child’s treatment-related information, and health care coverage.

#### Focus Groups

Subsequently, participants were scheduled for focus group sessions with peers from the same health region and age group (parents, adolescents aged 15-16 years, or adolescents aged 17-18 years). Focus group sessions were conducted between May 2022 and February 2023. Each focus group session lasted approximately 90 minutes and was conducted with 2-8 participants. The focus groups were facilitated by 2 female researchers with complementary expertise (more details are provided in the *Ensuring Rigor and Trustworthiness* section). The facilitators (TST or TIY) led groups using a focus group guide (Table 1), which was designed based on the study’s goals and existing literature on similar populations [4,14]. The focus group guide underwent a walk-through with the research team before data collection to ensure its clarity and relevance. This process allowed us to refine the questions, ensuring they were appropriate and aligned with the objectives of the study.

**Table 1:** Focus group guide: questions used to guide the adolescent and parent focus groups.

Focus groups	Questions
Adolescents	As someone living with T1D^a^, what kind of emotional or mental health support do you need? When you are frustrated with having T1D, who do you turn to for support? What topics or situations do you find yourself needing the most support for? How receptive would you be to getting support from other people with TID your own age? What are your thoughts about seeking support from slightly older people with T1D (ages 19-30 years)? What T1D-specific social media networks have you used before (Connected in Motion, JDRF^b^, Facebook groups, or any online communities)? Demo the REACHOUT App—then ask “What did you like about the REACHOUT App?”^c^ How important would it be to have health care professionals (ie, nurses, dieticians, psychologists) involved in REACHOUT NexGEN?
Parents	What do you worry about the most raising an adolescent with T1D? What kind of support do you need with regard to being a parent of an adolescent with T1D? When you are frustrated with T1D-related issues, who do you turn to for support? What topics or situations do you find you need the most support around? What are the issues or situations that you and your daughter/son have the most conflict about (related to T1D management)? What T1D-specific social media networks have you used before (Connected in Motion, JDRF, Facebook groups, or any online communities)?

^a^T1D: type 1 diabetes.

^b^JDRF: Juvenile Diabetes Research Foundation.

^c^We showed participants a video of the app. Afterward, we asked the questions “What did you like about the app?” and “How important would it be to include certain features?”

Adolescent and parent focus group questions differed slightly but focused on similar topics, with the adolescent focus groups viewing a demonstration of the existing REACHOUT app. While focus groups were structured with core and follow-up questions, organic discussions were encouraged to gain further insights and clarity on specific ideas. Focus groups were conducted online through Zoom videoconferencing software, and at the beginning of the session, the facilitator discussed the session rules and privacy and confidentiality.

The focus group size was designed to allow for diverse input while maintaining a manageable and comfortable setting. The target size was 4-6 participants per group, which is generally recommended in the literature to promote rich discussion while allowing everyone to participate [15]. However, due to logistical constraints, some groups were smaller than anticipated. While smaller groups may limit diversity of opinion, they may foster a more intimate and open environment, encouraging participants to share more personal insights. Combining groups from the same age range might have enhanced the diversity of viewpoints; however, separate groups were maintained based on scheduling and participant preferences, with the smaller groups offering a more personalized discussion.

The focus groups are intended to identify key user preferences and insights that will inform future redesign efforts. The insights from the focus group will form part of the revised requirements for the app redesign based on the adult app [12].

#### Data Processing and Analysis

Survey responses were analyzed using descriptive statistics using SPSS Statistics for MacBook (version 29.0; 2023; IBM Corp), with frequency data expressed as count (%) and continuous data expressed as mean (SD).

To ensure the accuracy of the focus group data, audio recordings of the focus groups were automatically transcribed by Zoom, deidentified by removing names, and then further verified by TIY. A thematic analysis of the resulting transcripts followed an inductive and iterative process to develop themes and subthemes [19]. Two coders, TIY and PJ, independently coded each of the transcripts using NVivo 12 (Lumivero). The coders compared their results throughout the coding process, ensuring consistency and accuracy. In cases of disagreement, coders reviewed the relevant data together and reassessed their coding decisions. If a consensus could not be reached, TST or MG made the final decision after reviewing the codes in the context of the research questions and focus group guide. After completing the first round of coding from the last focus group, we determined that thematic saturation had been reached and decided to end the recruitment and data collection process [20]. Finally, the study team convened to discuss and establish a unified codebook, organizing the identified themes and subthemes.

#### Ensuring Rigor and Trustworthiness

We ensured the rigor and trustworthiness of our research through triangulation, combining prestudy surveys and focus groups to capture diverse perspectives. Peer debriefing by 2 independent coders (TIY and PJ) validated themes, ensuring consistency and accuracy. Thematic saturation confirmed no new significant themes emerged.

We documented each research step to maintain transparency and reduce bias, enhancing credibility. The team’s positionality also strengthened the process: TT, with over 25 years of experience in qualitative methodologies, provided theoretical expertise; TIY, an MSc student with an MBBS, contributed clinical insights and methodological knowledge; and SP and PJ, a medical student and graduate, respectively, added relevant academic and practical experience.

## Results

### Participants

Out of 48 adolescents with T1D and 26 parents of adolescents living with T1D who expressed initial interest in the study, 16 of the former and 17 of the latter consented, and 10 of both groups participated in the focus group discussions. Reasons for nonparticipation (n=54) included the inability to reestablish contact after initial consent (28/54, 52%), loss of interest (7/54, 13%), scheduling conflicts (11/54, 20%), and “no show” to focus group session despite previous confirmation (8/54, 15%). Participants included parent-child dyads, parents without their children, and children without their parents.

### Prefocus Group Survey

The mean age of adolescent participants was 16 (SD 1) years and they had been living with diabetes for an average of 9 (SD 5) years (Table 2). Parent participants had a mean age of 51 (SD 7) years and were mostly (9/10, 90%) female (Table 3).

**Table 2:** Characteristics of adolescent participants (n=10).

Variables			Values
**Age (years), mean (SD)**			16 (1)
**Age at diagnosis (years), mean (SD)**			9 (5)
**Sex, n (%)**			
	Male	2 (20)	
	Female	8 (80)	
**Racial background, n (%)**			
	White	8 (80)	
	East Asian	1 (10)	
	Other	1 (10)	
**Insulin delivery system, n (%)**			
	Multiple daily injections	5 (50)	
	Insulin pump	5 (50)	
**Blood glucose monitoring device, n (%)**			
	Continuous glucose monitor	6 (60)	
	Flash glucose monitor	2 (20)	
	CGM^a^+lancets and strips	2 (20)	
**Continuous or flash glucose monitor type, n (%)**			
	Dexcom G6	8 (80)	
	Freestyle libre	2 (20)	
**Diabetes care provider, n (%)**			
	Endocrinologist	6 (60)	
	Family physician	2 (20)	
	Diabetes nurse	1 (10)	
	Other	1 (10)	

^a^CGM: continuous glucose monitoring.

**Table 3:** Characteristics of parent participants (n=10).

Variables			Values
**Age (years), mean (SD)**			51 (7)
**Sex, n (%)**			
	Male	1 (10)	
	Female	9 (90)	
**Racial background, n (%)**			
	Arab	1 (10)	
	White	9 (90)	
**Education, n (%)**			
	High school graduate	1 (10)	
	Some college or technical graduate	5 (50)	
	College graduate	1 (10)	
	Graduate or professional degree	3 (30)	
**Total household income (CAD $)^a^, n (%)**			
	$20,000-$29,999	1 (10)	
	>$90,000	9 (90)	
**Child’s insulin delivery system, n (%)**			
	Multiple daily injections	2 (20)	
	Insulin pump	8 (80)	
**Child’s blood glucose monitoring device, n (%)**			
	CGM^b^	6 (60)	
	Flash glucose monitor	2 (20)	
	CGM+Lancets and strips	2 (20)	
**Extended health care coverage, n (%)**			
	Yes	8 (80)	
	No	2 (20)	
**Counseling services coverage^c^, n (%)**			
	Child only	1 (13)	
	Family	2 (25)	
	No coverage	2 (25)	
	I don’t know	3 (38)	

^a^CAD $1 = US $0.76.

^b^CGM: continuous glucose monitoring.

^c^Only participants with extended health coverage (n=8) were asked this question.

### Focus Groups

We conducted 5 focus groups: 2 groups consisted of parents, with 1 group comprising 6 participants, and the other having 4 participants; the remaining 3 groups were composed of adolescents, with 1 group of 3 participants aged 15-16 years, another group of 5 participants aged 17-18 years, and the final group including 2 participants aged 15-16 years.

Four overarching themes were identified, with 3 themes exploring the support needs of adolescents living with T1D and 1 theme exploring their preferences for a peer-led mental health support app. These themes were (1) experience: navigating adolescence with T1D, (2) empowerment: support systems that enabled better management of their T1D, (3) obstacles: societal barriers that affect adolescent’s T1D management, and (4) innovation: adolescent-driven preferences for digital peer support platforms. These 4 themes were then further categorized into subthemes.

### Theme 1: Experience—Navigating Adolescence With T1D

Subthemes included (1a) challenges beyond physical health, (1b) balancing T1D management and independence in adolescent-parent relationships, and (1c) transitioning toward managing diabetes independently.

#### Subtheme 1a: Challenges Beyond Physical Health

Most adolescents described diabetes as a “lonely” condition and reported difficulty finding peers with the same emotional struggles. Adolescent concerns included fear of hypoglycemia in unfamiliar situations, anxiety about long-term complications, and challenges of everyday activities such as driving or writing exams. Even when feeling anxious, some adolescents were still reluctant to discuss these worries with health care professionals, family, and friends.

I don’t have anyone to talk to, and I just like to go through it, which probably adds a lot more stress to me having to be all alone going through that.

Adolescent 1

Adolescents also described specific instances where they felt isolated, such as during school trips or exams, when managing diabetes became a visible and misunderstood challenge among peers.

My friends don’t get why I always carry snacks or why I sometimes leave during class—it makes me feel different and not in a good way.

Adolescent 3

Parents expressed different concerns, such as shielding their children from worry while encouraging responsible diabetes management.

I don't want to scare her into worrying about, you know, potential problems with losing limbs or heart attacks or strokes, or the absolute worst possible things.

Parent 4

#### Subtheme 1b: Balancing T1D Management and Independence in Adolescent-Parent Relationships

Encouraging adolescents to prioritize diabetes care created a complicated dynamic between adolescents and parents. While parents wanted to instill a sense of responsibility in their children, they did not want to be perceived as overbearing (ie, “helicopter parents”).

I find that there was a period where my son would systematically forget to bolus for his meals, and as a parent, I just had to nag him and nag him, and I think that hurt our relationship.

Parent 3

Communication with parents was particularly challenging when adolescents felt overwhelmed by constant reminders and pressure regarding management.

I don't want to bring up my care and then have them like be more stressed and be on me more because their way of supporting me is like bugging me.

Adolescent 9

However, parents also mentioned that when they engaged in constructive communication, they improved their relationship with their children.

#### Subtheme 1c: Transitioning Toward Managing Diabetes Independently

As adolescents approached adulthood, some parents recognized the need to relinquish some diabetes-related responsibilities and shift them onto their children.

There definitely was a transition period where I had to let him take over, and it wasn't perfect. In fact, it was a scary thing to do, but I find that eventually, by backing away and letting him take charge, he did take charge, and he's much, much better today.

Parent 5

This sentiment was echoed by several adolescents, particularly those traveling far from home to attend university.

I’m going away for university next year, and I feel like it’s because my parents—I’ve kind of been able to prove to my parents that I can be independent, but I was doing that by kind of like trial and error.

Adolescent 1

In contrast, other adolescents were not ready to assume complete management control and chose to remain at home close to their parents.

### Theme 2: Empowerment—Support Systems That Enable Better Management of Their T1D

Subthemes included (2a) the role of online support systems, (2b) family and community support as foundational support systems, and (2c) interest in peer connections.

#### Subtheme 2a: Role of Online Support Systems

Both adolescents and parents discussed the value of online support systems for connecting with others living with T1D or caring for a child with T1D. These platforms helped reduce isolation and foster companionship with individuals who understood their experience. Adolescents highlighted the importance of online communities to share experiences with peers managing T1D, while parents appreciated the role of these communities in providing access to advice from other caregivers.

I find that if I look online, and I see discussion of other people and their struggles with diabetes, I feel a little bit less lonely, but it still isn't quite the same as having someone to talk to and relate to.

Adolescent 10

I have found some Facebook support groups, and I’ve been looking at them, and in many ways, some of them I have vented on there, and I have learned a lot.

Parent 4

While these digital environments offered the space to exchange thoughts and frustrations about T1D, some adolescents found these online groups overwhelming, primarily when discussions evolved into emotion-heavy topics such as long-term complications.

#### Subtheme 2b: Family and Community as Foundational Support Systems

Family members were described as the cornerstone of support. Adolescents noted that siblings often stepped in to help with reminders or provided companionship during health care appointments. Parents, on the other hand, saw themselves as “safety nets,” providing structure to daily management tasks.

If I have any new issues that I realized have come up that I need help Problem Solving, my mom is definitely my go-to person since she knows the situation well.

Adolescent 8

Although many adolescents leaned on parents and friends for support, talking about diabetes with loved ones was not always satisfying. Instead, some adolescents valued connecting with other T1D peers who could offer empathy and understanding and exchange practical information.

Parents accessed community support by connecting with other parents of children with T1D and exchanging tips and information.

It's super important to feel supported and just be able to have another mom say to you, oh, this is where you get this, this is where you get the small juice boxes that, you know, all the little tips and tricks that.

Parent 3

#### Subtheme 2c: Interest in Peer Connections

Adolescents expressed a strong desire to connect with young adults with T1D (ie, near-peers) who have successfully achieved independence in managing their diabetes, while parents echoed this need from their perspective, hoping to reduce adolescents’ feelings of loneliness.

It would be nice to talk to someone my age who gets it—like what it’s like to have T1D during a school trip or stuff like that.

Adolescent 5

I want to know how older diabetics are achieving independence and what role diabetes plays in their life.

Parent 8

They sought insights on managing diabetes in the work and school setting. Parents were equally eager to help their children link up with relatable peers to reduce feelings of loneliness and isolation during a challenging period in life.

I'm looking also for her to find peers who have a similar medical condition so that she doesn’t feel like she’s so alone as a teenager.

Parent 7

### Theme 3: Obstacles—Societal Barriers That Affect T1D Management

Subthemes included (3a) insurance-related obstacles, (3b) stigma and discrimination surrounding diagnosis, and (3c) lack of understanding by the public.

#### Subtheme 3a: Stigma and Discrimination Surrounding Diagnosis

Adolescents recounted situations where they felt unfairly scrutinized by authority figures, such as being accused of using illicit drugs (use of needles for insulin administration). Stigma and discrimination often originated from individuals outside the immediate family and peer group; however, these negative comments sometimes also came from friends. Anticipating these situations, many patients and families guarded their diagnosis from others. Those who disclosed their condition often found themselves mistaken for having type 2 diabetes and were targets of pejorative stereotypes (eg, poor lifestyle habits).

You’ve probably seen like hundreds of jokes that are like, oh, if you eat that, you’re gonna get diabetes and then, of course, that makes you feel bad because there’s that stigma, and that’s just so not true.

Adolescent 9

Some parents reported concealing their child’s diagnosis until it was essential to disclose it, such as when starting a new job.

It wasn’t easy for my son to get a job because of, you know, the circumstances around his health.

Parent 3

Because of the stigma around diabetes, parents needed to advocate for their children in the school and work setting and encourage them to advocate for themselves.

#### Subtheme 3b: Insurance Coverage Obstacles

Participants also expressed frustrations with navigating insurance coverage.

I don’t understand how insurance works. I don’t know how they’re going to cover the cost of my diabetes supplies. I don’t know what they cover; I don’t have that information.

Adolescent 9

For many parents, securing lifesaving supplies for their children was an arduous process that involved hours on the phone.

It takes two hours and three hours of my day to stay on top of things and get updates from insurance companies and stay on hold, and all of this, and I feel like if there’s a shortcut of information, that would be amazing.

Parent 3

They also invested considerable time on the internet searching for pertinent information. Adolescents reported a general lack of knowledge regarding the policies and procedures of medical insurance and the costs of diabetes supplies. Not surprisingly, these frustrations were noted by participants preparing to leave home to attend university.

#### Subtheme 3c: Lack of Understanding by the Public

Both adolescents and parents reported negative experiences when talking to the general public about diabetes. Not only did individuals without T1D make inaccurate assumptions and demonstrate a lack of knowledge and sensitivity, but also these conversations often required exhausting explanations and effort.

Explaining it to somebody creates more work for the diabetic than it does to help them because, first, you need to explain it before, and you can tell them what’s bothering you about it, so they understand how everything works. So, it creates more work, so sometimes it’s easier just to not open up the conversation.

Adolescent 1

Consequently, participants avoided situations or interactions where the topic of diabetes could emerge.

It’s pointless to go to others because I would have to teach them, so my daughter and I just talk about it amongst ourselves.

Parent 4

### Theme 4: Innovation—Adolescent-Driven Preferences for Digital Peer Support Platforms

Subthemes included (4a) information security and accuracy, (4b) enhancing user interface and user experience, and (4c) add-ons for optimizing interactions.

#### Subtheme 4a: Information Security and Accuracy

Many participants expressed concern about exchanging inaccurate and potentially harmful medical advice about insulin pumps, dosages, dietary restrictions, and so on.

Information about managing your pump and insulin, bolusing, and how you will lose a dress size in a matter of two weeks, there’s a lot of curiosity around that, so I'm concerned about that.

Parent 4

One suggestion to reduce this risk was to have health care professionals moderate group chats.

I think there could be some privacy issues, and what can be talked about. I don’t know if it’s kind of overkill but there could be like mediators, especially on chats that might be covering more sensitive topics.

Adolescent 9

#### Subtheme 4b: Enhancing the User Interface and User Experience

Participants expressed positive feedback about the planned REACHOUT NexGen platform, appreciating its concept, components (group chat, personal messaging, and access to trained near-peer mentors), and potential benefits for peer discussions. Parents wanted the platform to be user-friendly, straightforward, and enjoyable for adolescents, while adolescents focused more on aesthetics, user experience, and navigation assistance. Specifically, they suggested a more welcoming color palette and an introductory tutorial guiding users through its various components.

For the like homepage sort of thing, it looks kind of like intense, like it looks like Microsoft teams, which is kind of like intimidating.

Adolescent 1

Just like the design of the homepage a little bit maybe. I don’t know, I’m not good with design, but maybe it could change a little bit just to make it look more visually appealing.

Adolescent 2

#### Subtheme 4c: Add-ons for Optimizing Interactions

Participants recommended features to incorporate into an ideal digital support platform, such as the ability to pin messages or chats on the platform’s home screen and complete phone calls or video calls on the platform. These features, currently absent in the adult version of REACHOUT, were proposed to enrich user interactions and connectivity.

You know in iMessage, for example, you are able to pin a certain conversation, so it becomes like a bubble at the top of your list, so it’s like a priority almost. [Adolescent 7]

If there could be video calls or even phone calls, it would be nice, so you don’t have to get off the app if you want to speak to someone on the phone. [Adolescent 1]

## Discussion

### Principal Findings

This study explored the support needs of adolescents with T1D, focusing on the psychosocial challenges they face during an already demanding stage of life characterized by puberty-related changes, academic pressures, peer dynamics, and increased conflict with parents. In doing so, it provided specific design insights for app-based peer support, including features such as moderated chats for safety and video calls to foster emotional connection. These findings address gaps in the literature by demonstrating how technology can be tailored to meet adolescents’ unique support needs and highlight ways to adapt an existing app (T1D REACHOUT), initially designed for adults, to better serve the adolescent population with T1D.

### Comparison With Previous Work

Other studies [4,21,22] have also observed concerns about diabetes management, fear of long-term complications, strained relationships with parents, and transition into adulthood. For example, Castensøe-Seidenfaden et al [21] identified key worries among 9 adolescents aged 15-19 years and 13 parents, including safety in managing diabetes, independence, and apprehensions about future health complications.

Our results also revealed the pivotal role of support systems. Over and above family support, which has been shown to have a positive impact on mental health in adolescents with T1D [23,24], our participants voiced a clear desire to connect with peers with T1D. As adolescents approach adulthood, they gravitate more strongly toward their friends for support rather than their parents [25], as noted in subtheme 1b, where adolescent participants expressed being overwhelmed by their parents. In the context of T1D, peer support offers a space to exchange viewpoints and experiences regarding specific challenges, foster mutual understanding, and encourage collaborative problem-solving [26,27].

Furthermore, engaging in peer activities bolsters adolescents’ capacity for empathy and support [28]. It can play a significant role in alleviating stress and anxiety during times of transition, as noted in subtheme 2c, where adolescent participants expressed desires to connect with peers and near-peers. Previous research among adolescents with T1D has found a link between peer support and improved diabetes outcomes. For example, Doe [29], in a study of 90 adolescents aged 15-18 years, observed a significant association between peer support and better glycemic control. Similarly, in a study by Raymaekers et al [30] involving a large cohort of 467 individuals, including adolescents (14-17 years) and emerging adults (18-25 years), it was found that increased emotional support from peers predicted lower levels of diabetes-related distress.

Our findings also highlight the specific ways adolescents wish to connect with peers with T1D, such as through moderated digital platforms that enable both group and one-on-one interactions. This expands upon previous work by Doe [29], which linked peer support with better glycemic control but did not explore the exact mechanisms or features adolescents preferred for peer interactions. Finally, our data provide insights that inform the design and implementation of a peer-delivered mental health support mobile app for adolescents. Using the principles of human-centered design [31], we were able to transform insights from theme 4 into actionable design strategies for our app; this included refining the app through a streamlined interface, clear color schemes, clutter reduction, user tutorials, message pinning, enhanced connectivity through calls, and moderated chats for safety. Although the integration of features such as phone and video calls has predominantly been used to provide support between scheduled visits with the diabetes care team and to facilitate online clinic appointments with health care providers [32], our findings suggest that these modes of communication may also foster a sense of companionship and emotional connection with peers. Similarly, other studies have identified app-related preferences for this T1D age cohort, such as user-friendliness, ease of navigation, and safe participation by moderating peer discussions [13,14,33]. For example, the self-compassion chatbot (called “COMPASS”) app [33], designed for adolescents aged 12-16 years with T1D, demonstrated improvements in psychosocial well-being among adolescents with T1D, but participants in our study advocated for safe discussions with their peers and features that can assist in easy navigation, such as a search bar function.

While there are existing platforms that adolescents with T1D have already leveraged to obtain peer support (eg, Reddit, Discord, and TikTok), these online environments lack two core features: (1) access to focused one-on-one support delivered by a trained near-peer and (2) health care professional–monitored chat rooms and discussion boards [34]. Over and above same-age peers, adolescents have expressed a desire for support from young adults with T1D who have more years of life experience to share [4]. Furthermore, adolescents seek security in knowing safeguards are in place to prevent the exchange of medically contraindicated information [35]. In response to this gap, our platform, REACHOUT NexGEN, will incorporate these critically important features. For example, T1D REACHOUT, the adult version of the app, uses trained moderators and health care professionals to oversee chat rooms and discussion boards, ensuring that the information exchanged is accurate and safe [12]. This moderation system helps protect users from receiving inaccurate advice, a concern that was echoed in our study by adolescent participants who emphasized the importance of safeguards. By adopting these practices, REACHOUT NexGEN will offer a safe and secure space for adolescents with T1D to connect with both peers and near-peers, therefore addressing their need for support while safeguarding their well-being.

### Limitations

This study has limitations. First, one of our focus groups (adolescents aged 15-16 years) had only 2 participants, which may have hindered in-depth discussion; however, we obtained some useful points from the discussion, and we ran another focus group with more participants from this age group. Second, most of our participants were female and may have been more inclined to openly discuss health issues [36] and engage in research studies [37]. The majority of the participants were female, which may have influenced the support needs emphasized in our findings. Female adolescents are often more likely to articulate psychosocial challenges and emotional well-being, which may have led to a stronger focus on these areas [36]. Female adolescents with T1D often experience higher levels of DD due to a combination of hormonal fluctuations once they reach menarche, which complicates blood glucose management and psychosocial factors, including body image concerns and increased risk of eating disorders [38]. Conversely, the lack of male representation may mean that certain challenges, such as stigma around discussing diabetes among male peers or unique preferences for technological interactions, were underrepresented. A more gender-diverse sample could provide a more balanced perspective on the support needs of the broader adolescent population with T1D. Also, our focus group participants lacked sociodemographic diversity and may not reflect the larger adolescent population with T1D [39]. Finally, the variation in focus group sizes, influenced by participant preferences and scheduling constraints, may have limited broader discussions and diversity of perspectives. While smaller groups fostered personalized interactions, future studies should aim to balance participant preferences with recommended group sizes to enhance discussion dynamics.

These factors all potentially limit the generalizability of our findings. Future studies should explore strategies to engage a more heterogeneous sample by actively collaborating with community organizations, advocacy groups, or cultural associations representing various demographic groups, as this could contribute to a more nuanced understanding of the complexities within different demographic groups.

### Conclusions

This study confirmed the existing and compelling evidence of the need for mental health support for adolescents with T1D. It also showed that adolescents are interested in the potential benefits of app-based peer support for providing emotional assistance. Further research is required to evaluate the platform’s feasibility and effectiveness to uncover potential challenges, refine design features based on user feedback, assess user engagement and satisfaction, and evaluate the app’s sustained impact over time.

## Appendix

Multimedia Appendix 1 COREQ (Consolidated Criteria for Reporting Qualitative Research) checklist.

Multimedia Appendix 2 T1D REACHOUT NexGEN Study: adolescent focus group participant questionnaire.

Multimedia Appendix 3 T1D REACHOUT NexGEN Study: focus group parent questionnaire.

## Abbreviations

BCCH: BC Children’s Hospital
CGM: continuous glucose monitor
COREQ: Consolidated Criteria for Reporting Qualitative Research
DD: diabetes distress
IHA: Interior Health Authority
REDCap: Research Electronic Data Capture
T1D: type 1 diabetes
VIHA: Vancouver Island Health Authority

## Data Availability

The datasets generated and analyzed during this study are not publicly available due to the privacy and confidentiality requirements of the research ethics board.
