# Supplementary material for: Exploring the Needs and Preferences of Users and Parents to Design a Mobile App to Deliver Mental Health Peer Support to Adolescents With Type 1 Diabetes: Qualitative Study
Source: JMIR Diabetes. 2025 Jan 20;10:e64267. doi: 10.2196/64267 (PMC11791445; doi:10.2196/64267)
Supplement: Multimedia Appendix 2 [file diabetes_v10i1e64267_app2.docx]

**T1D REACHOUT NexGEN Study: Adolescent Focus Group Participant Questionnaire**

**SECTION A: DEMOGRAPHICS**

**Please answer every question by filling in the blank(s), circling the correct answer, or checking the correct box(s). If there is any question you do not wish to answer, please select ‘Decline to Answer’.**

1. What is your age? __ __ years old
2. What is your biological sex?: _1_ Male _2_ Female _3_ Other ___________ _4_ Decline to Answer
3. Which country were you born in? ____________________________
4. If you were not born in Canada, how long have you lived in Canada? ________
5. How old were you when you were first told you had diabetes? __ __
6. How many siblings are currently living at your home?

- 1 sibling
- 2 siblings
- 3 or more siblings

1. What is your ethnic origin? (check all that apply)

_1_ Aboriginal

_2_ Arabic

_3_ East Asian (Chinese, Korean, Japanese)

_4_ South Asian (Bangladesh, Pakistan, Indian)

_5_ Southeast Asian (Vietnamese, Cambodian)

_6_ White

_7_ Native Hawaiian or other Pacific Islander

_8_ Black

_9_ Other _______________

_10_ Decline to Answer

**SECTION B: DIABETES STATUS AND HEALTH CARE**

**Please answer every question by filling in the blank(s), circling the correct answer, or checking the correct box(s). If there is any question you do not wish to answer, please select ‘Decline to Answer’**

1. Which form of insulin delivery do you currently use?: (check all that apply)

_1_ Multiple Daily Injections

_2_ Insulin Pump

_3_ Both

_4_ Neither (if neither then please specify form)

_5_ I don’t know

_6_ Decline to Answer

1. If you use an insulin pump, which insulin pump do you currently use?

_1_ Omnipod

_2_ Medtronic 670G

_3_ Medtronic 630G

_4_ Minimed Paradigm 530G

_5_ Tandem T:Slim

_6_ Animas OneTouch Ping

_7_ Animas Vibe

_8_ YpsoPump

_9_ Other: ___________

_10_ I don’t know

_11_ Decline to Answer

I don’t know

1. What do you use to monitor your blood glucose levels?

_1_ Continuous glucose monitor (CGM)

_2_ Flash Glucose Monitor (Freestyle Libre)

_3_ Home blood glucose monitoring with lancets and test strips (ie finger pokes with a home blood glucose monitoring device).

_4_ Not Applicable

_5_ I don’t know

_6_ Decline to Answer

1. If you use a CGM, which continuous glucose monitor (CGM) do you currently use?

_1_ DexCom 6G

_2_ DexCom 5

_3_ DecCom 4

_4_ Medtronic Guardian

_5_ Other ____

_6_ I don’t know

_7_ Decline to Answer

1. Do you take any other diabetes medications or injectables in addition to insulin? Please check all that apply:

_1_ Canagliflozin (Invokana)

_2_ Dapagliflozin (Farxiga)

_3_ Empagliflozin (Jardiance)

_4_ Dulaglutide (Trulicity)
_5_ Exenatide  (Byetta)

_6_ Exenatide ER (Bydureon)

_7_ Liraglutide (Victoza)

_8_ Lixisenatide (Adlyxine)

_9_ Semaglutide (Ozempic)

_10_ Liraglutide + Insulin Degludec (Xultophy)

_11_ Lixisenatide + Insulin Glargine (Soliqua)

_12_ Other(s): _____________

_13_ I don’t know

_14_ Decline to Answer

1. Which health care provider(s) do you currently have?

_1_ Endocrinologist

_2_ GP

_3_ Diabetes nurse

_4_ Dietitian

_5_ Pharmacist

_6_ Other: ____________

_7_ Decline to Answer

1. Which health care provider helps you the most with managing diabetes?

_1_ My endocrinologist

_2_ My GP

_3_ My diabetes nurse

_4_ My dietitian

_5_ My pharmacist

_6_ Other: ____________

_7_ Decline to Answer

1. Who has been your primary diabetes care provider for the past 6 months?

___________________

**SECTION C: PEER SUPPORTER AND TECHNOLOGICAL PREFERENCES**

**Please answer every question by filling in the blank(s), circling the correct answer, or checking the correct box(s). If there is any question you do not wish to answer, please select ‘Decline to Answer’**

1. T1D NexGEN will include a library of peer mentors for users to choose from. A peer mentor is an adult with T1D who has completed training in active listening, basic motivational interviewing skills, and behavior modification techniques. If you had to select a Peer Mentor you want to be matched with, what would you want to know about them before you pick one? Check all that apply

_1_ Gender

_2_ Age

_3_ Ethnicity/ Cultural background

_4_ Place of residence (city)

_5_ Age at T1D diagnosis

_6_ Number of years living with T1D

_7_ Multiple Daily Injections or Pump

_8_ CGM

_9_ FGM

_10_ Do-it-yourself looping

_11_ Involvement in team sports or other forms of intense physical activity

_12_ Hobbies and interests (e.g. cooking, technology, traveling, etc.)

_13_ Diet regime (e.g. keto, paleo, plant-based, gluten-free)

_14_ Personality traits (e.g. extroverted/introverted, organized, open-minded,

friendly, sensitive)

_15_ Others: _______________

_16_ Decline to Answer

1. Which social media platforms do you use on a regular basis?

_1_ Facebook

_2_ Reddit

_3_ Twitter

_4_ Instagram

_5_ Others: ________________

_6_ Decline to Answer

1. Which social media platforms do you use to access diabetes support? If you use a particular platform, please enter the name of the specific group(s)/account(s) you use/follow.

_1_ Facebook

Group/Account name(s): ______________

_2_ Reddit

Subreddit name(s):___________

_3_ Twitter

Account name(s):___________

_4_ Instagram

Account name(s):___________

_5_ TikTok

Account name(s):____________

_6_ Snapchat

Account name(s):______________

_7_ Others: ________________

_8_ None, I don’t access diabetes support on social media

_9_ Decline to Answer

1. What diabetes specific T1D-specific social networks are you involved in?

_1_ Beyond Type 1

_2_ Diabetes Canada

_3_ JDRF

_4_ T1D Huddle

_5_ Others: ________________

_7_ None, I’m not involved in any T1D-specific social networks

_6_ Decline to Answer

21) Below is a list of topics that may be of concern to you as someone living with T1D. We want you to rank which topics are most significant to you.

To accomplish this, we are going to perform a thought experiment. Imagine we gave you $100. Using this $100, you will place a certain amount of money on topics that you find most significant. The amount of money you place should be relative to how significant you think that topic is.

For example, if you only think two topics in this list are significant and they are equally significant, you may give each one $50. Another example: let’s say you think only “Topic 1” is very significant, and “Topic 2,” and “Topic 3” are moderately significant, and the rest don’t matter to you- then you may choose to split up the $100 into $50, $25, $25 for the three topics.

You can split it up however you want and in whatever quantity based on how important you think each section is. You can give $0 if you think a section isn’t important at all.

In short, we want you to assign the relative value of each of these topics by spending an imaginary $100. Just remember you get max $100. If you’re confused about this, please feel free to contact a team member to explain!

Please rank the topics that are of most concern to you as someone living with T1D (Max $100)

| # | Field |
| --- | --- |
| 1 | My day-to-day management |
| 2 | How I can best support my diabetes care |
| 3 | Communicating about T1D |
| 4 | Worries about my future (both health and other aspects) |
| 5 | Parents-child conflicts with regard to T1D management |
| 6 | How to cope with constant fear and anxiety that I may go low when away from home |
| 7 | Feeling incompetent, distressed, and burned out |
| 8 | How to become more independent with regard to T1D management |
| 9 | Financial strain associated with T1D supplies |
| 10 | Impact of T1D on my emotional wellbeing and mental and health |
| 11 | New technologies in the management of T1D |
| 12 | Social and peer pressure |
