# Supplementary material for: Exploring the Needs and Preferences of Users and Parents to Design a Mobile App to Deliver Mental Health Peer Support to Adolescents With Type 1 Diabetes: Qualitative Study
Source: JMIR Diabetes. 2025 Jan 20;10:e64267. doi: 10.2196/64267 (PMC11791445; doi:10.2196/64267)
Supplement: Multimedia Appendix 3 [file diabetes_v10i1e64267_app3.docx]

**T1D REACHOUT NexGEN Study: Focus Group Parent Questionnaire**

**Please answer every question by filling in the blank(s), circling the correct answer, or checking the correct box(s). If there is any question you do not wish to answer, please select ‘Decline to Answer’.**

**SECTION A: DEMOGRAPHICS**

1. What is your sex?

- Female
- Male
- Other, please specify
- Decline to Answer

2. Please report your *ethnicity* by selecting all that apply:

_1_ Aboriginal

_2_ Arabic

_3_ East Asian (Chinese, Korean, Japanese)

_4_ South Asian (Bangladesh, Pakistan, Indian)

_5_ Southeast Asian (Vietnamese, Cambodian)

_6_ White

_7_ Native Hawaiian or other Pacific Islander

_8_ Black

_9_ Other _______________

_10_ Decline to Answer

3. What is your total household income?

- <$20,000
- $20,000 to $29,999
- $30,000 to $39,999
- $40,000 to $49,999
- $50,000 to $59,999
- $60,000 to $69,999
- $70,000 to $89,999
- ≥$ 90,000
- Decline to Answer

4. What is your highest level of education?

☐ Less than high school

☐ High school graduate (or equivalent)

☐ Some college or technical school

☐ College graduate

☐ Graduate or Professional Degree(s)

☐ Decline to Answer

**Section B: DIABETES CARE**

| 5 a. Do you have extended health coverage for your child?  ☐ Yes  ☐ No  ☐ Decline to Answer  5 b. [IF 5A IS YES] If yes, please provide the name of insurance company your child receives the extended health coverage through?  5c. [IF 5A IS YES] On average, how much time per month do you spend talking with your extended health carrier about coverage for any T1D related treatment or devices?  ☐ 0-1 hour per month  ☐ 1-2 hours per month  ☐ 3 or more hours per month  ☐ Decline to Answer  6.Which form of insulin delivery does your child currently use? (check all that apply)   - _1_Multiple Daily Injections - _2_Insulin Pump - _3_Both - _4_Neither ______ (if neither then please specify form) - I don’t know - _5_Decline to Answer   7. What does your child use to monitor your blood glucose levels?   - _1_Continuous glucose monitor (CGM) - _2_Flash Glucose Monitor (Freestyle Libre) - _3_Home blood glucose monitoring with lancets and test strips (ie finger pokes with a home blood glucose monitoring device). - _4_Not Applicable - I don’t know - _5_Decline to Answer   8a. [IF 5A IS YES] Does your health plan cover your child’s insulin?  ☐ No Coverage  ☐ Partial Coverage  ☐ Full Coverage  ☐ I don’t know  ☐ Decline to answer  8b. [ONLY IF 8a = PARTIAL COVERAGE] If partial coverage is selected for insulin, please provide coverage percent  9a. [IF 5A IS YES AND 6 IS 1,2,3,4] Does your health plan cover your child’s insulin delivery method?  ☐ No Coverage  ☐ Partial Coverage  ☐ Full Coverage  ☐ I don’t know  ☐ Decline to answer  9b. [ONLY IF 9a = PARTIAL COVERAGE] If partial coverage is selected for insulin delivery method, please provide coverage percent  9c. [IF 5A IS YES AND 7 IS 1, 2, or 3] Does your health plan cover your child’s monitoring method?  ☐ No Coverage  ☐ Partial Coverage  ☐ Full Coverage  ☐ I don’t know  ☐ Decline to answer  9d. [ONLY IF 9C = PARTIAL COVERAGE] If partial coverage is selected for monitoring method, please provide coverage percent  10. [IF 5A IS YES] Does your health plan cover counselling services (select all that apply)?  ☐ For your child  ☐ For you and your partner  ☐ No Coverage  ☐ I don’t know  ☐ Decline to answer  **Section C: Experience**  11) Below is a list of topics that may be of concern to you as a parent of a child living with T1D. We want you to rank which topics are most significant to you.  To accomplish this, we are going to perform a thought experiment. Imagine we gave you $100. Using this $100, you will place a certain amount of money on topics that you find most significant. The amount of money you place should be relative to how significant you think that topic is.  For example, if you only think two topics in this list are significant and they are equally significant, you may give each one $50. Another example: let’s say you think only “Topic 1” is very significant, and “Topic 2,” and “Topic 3” are moderately significant, and the rest don’t matter to you- then you may choose to split up the $100 into $50, $25, $25 for the three topics.  You can split it up however you want and in whatever quantity based on how important you think each section is. You can give $0 if you think a section isn’t important at all.    In short, we want you to assign the relative value of each of these topics by spending an imaginary $100. Just remember you get max $100. If you’re confused about this, please feel free to contact a team member to explain!  Please rank the topics that are of most concern to you as a parent of a child living with T1D (Max $100)   \| # \| Field \| \| --- \| --- \| \| 1 \| My child’s day-to-day management \| \| 2 \| How I can best support my child with their diabetes care \| \| 3 \| Communicating with my child about T1D \| \| 4 \| Worries about my child’s future (both health and other aspects) \| \| 5 \| Parents-child conflicts with regard to T1D management \| \| 6 \| How to cope with constant fear and anxiety that your child may go low when away from home \| \| 7 \| Feeling incompetent, distressed, and burned out \| \| 8 \| How to help my child become more independent with regard to T1D management \| \| 9 \| Financial strain associated with T1D supplies \| \| 10 \| Impact of T1D on emotional well being and mental and health \| \| 11 \| New technologies in the management of T1D \| \| 12 \| Social and peer pressure \|   12) What kind of support do you need as a parent of a child living with T1D? |
| --- | --- | --- | --- | --- | --- | --- | --- | --- | --- | --- | --- | --- | --- | --- | --- | --- | --- | --- | --- | --- | --- | --- | --- | --- | --- | --- |
|  |
